# Proinflammatory cytokine production and insulin sensitivity regulated by overexpression of resistin in 3T3-L1 adipocytes

**DOI:** 10.1186/1743-7075-3-28

**Published:** 2006-07-19

**Authors:** Yuchang Fu, Liehong Luo, Nanlan Luo, W Timothy Garvey

**Affiliations:** 1 Department of Nutrition Sciences, University of Alabama at Birmingham, Birmingham AL 35294–3360, USA; 2 Birmingham Veterans Affairs Medical Center, Birmingham AL 35233, USA

## Abstract

Resistin is secreted from adipocytes, and high circulating levels have been associated with obesity and insulin resistance. To investigate whether resistin could exert autocrine effects in adipocytes, we expressed resistin gene in 3T3-L1 fibroblasts using a lentiviral vector, and selected several stably-transduced cell lines under blasticidin selection.

We observed that 3T3-L1 adipocytes expressing resistin have a decreased gene expression for related transcriptional factors (CCAAT/enhancer binding protein α(C/EBPα) , peroxisome proliferator-activated receptor gamma (PPARγ), and adipocyte lipid binding protein (ALBP/aP2) which is one of target genes for the PPARγ during adipocyte differentiation,. Overexpression of resistin increased the levels of three proinflammatory cytokines, tumor necrosis factor alpha (TNFα), interleukin 6 (IL-6) and monocyte chemoattractant protein-1 (MCP-1), which play important roles for insulin resistance, glucose and lipid metabolisms during adipogenesis. Furthermore, overexpressing resistin in adipocytes inhibits glucose transport 4 (GLUT4) activity and its gene expression, reducing insulin's ability for glucose uptake by 30 %.

In conclusion, resistin overexpression in stably transduced 3T3-L1 cells resulted in: 1) Attenuation of programmed gene expression responsible for adipogenesis; 2) Increase in expression of proinflammatory cytokines; 3) Decrease in insulin responsiveness of the glucose transport system. These data suggest a new role for resistin as an autocrine/paracrine factor affecting inflammation and insulin sensitivity in adipose tissue.

## Background

Insulin resistance is a characteristic feature of Metabolic Syndrome and Type 2 Diabetes, and involves target tissues such as fat, liver, and skeletal muscle. The pathogenic mechanisms that impair insulin action in these tissues, and the factors responsible for the development of the Metabolic Syndrome trait cluster have not been fully elucidated. However, over the past decade, it has become clear the adipose tissue plays a central role in these processes. Adipocytes secret numerous factors, collectively referred to as adipocytokines, which circulate in blood and act on distal tissues to influence food intake, energy expenditure, and carbohydrate and lipid metabolism [1,2]. However, there is a relative paucity of data regarding mechanisms regulating adipocytokine secretion in adipose tissue. Resistin is an example of an important adipocyte secreted protein [3,4], and elevated resistin levels in adipose tissues and serum are observed in both genetic and diet-induced obesity and insulin resistance in animal models [5,6]. Resistin administration or hyperresistinemia impairs glucose tolerance and induces hepatic insulin resistance [7,8], whereas mice deficient in resistin are protected from obesity-associated insulin resistance [9]. Hence, resistin has been proposed as a link between obesity, insulin resistance, and hyperglycemia.

Our understanding of resistin's role in metabolism has advanced primarily by studying its direct effects on skeletal muscle and liver tissues and related cultured cell systems, while less is understood regarding autocrine/paracrine effects of resistin in regulating adipocyte biology and adipocytokine secretion. To address this question, we established stably transduced 3T3-L1 fibroblast cell lines using a lentiviral vector to hyperexpress resistin. We observed that overexpression of resistin impairs the insulin-stimulable glucose transport system by suppressing GLUT4 expression and modulates the secretion of inflammatory cytokines via an autocrine/paracrine mechanism.

## Methods

### Reagents

Mouse 3T3-L1 fibroblast cells were purchased from American Type Culture Collection (Manassas, VA). Tissue culture media were purchased from Life Technologies (Gaithersburg, MD). Insulin, dexamethasone (Dex) and isobutyl-methylxanthine (IBMX) were purchased from Sigma (St. Louis, MO). LacZ staining kit was purchased from Stratagene (San Diego, CA). RNA isolation solution was purchased from Biotecx Laboratory (Houston, TX). Horseradish peroxidase (HRP)-conjugated antibodies to the V5 epitope were purchased from Invitrogen (Carlsbad, CA), resistin antibody from Chemicon International (Temecula, CA) and TNFα, IL-6, IL-10 and MCP-1 antibodies from Santa Cruz Biotechnology (Santa Cruz, CA). 2-deoxy-D-[^3^H] and L-[1-^3^H] glucose were purchased from Amersham (Arlington Heights, IL). Unless otherwise specified, all other reagents were purchased from Sigma.

### Recombinant lentiviruses and lentiviral transduced cell lines

Fusion cDNAs, containing the full length of mouse resistin coding sequence and a V5 epitope tag, were cloned into a ViraPower-CMV vector (Invitrogen). The recombinant lentiviral plasmids and a control *LacZ *gene construct were transfected into HEK293 cells. Western blot and X-gal staining were performed to confirm that the HEK293 cell transfection was successful and infectious virus particles were produced. To establish stable 3T3-L1 cell lines which express resistin or LacZ genes, recombinant resistin or LacZ lentiviral stocks were used to infect 3T3-L1 cells with Polybrene (Specialty Media, Phillipsburg, New Jersey) at a final concentration of 6 μg/ml. Forty-eight hours post-transduction, these cells were placed under blasticidin selection (10 μg/ml) for 20 days. Western blot analyses were performed to test for stable resistin or LacZ gene expression in the selected cell lines after antibiotic selection.

### Cell culture and stimulation

3T3-L1 fibroblasts or transduced cell lines were grown and differentiated into adipocytes in 100-mm culture dishes, as described by Frost and Lane [10]. Briefly, cells were grown to 100% confluence in Dulbecco's minimal essential medium (DMEM) containing 25 mM glucose and 10% calf serum at 37°C in a humidified atmosphere containing 5% CO_2_. Two days after full confluence, cells were differentiated via incubation in DMEM containing 25 mM glucose, 0.5 mM isobutylmethylxanthine (IBMX), 1 μm dexamethasone (Dex), 10 μg/ml insulin, and 10% FBS for 3 days and then for 2 days in DMEM containing 25 mM glucose, 10 μg/ml insulin, and 10% FBS. Thereafter, cells were maintained in and refed every 2 days or 3 days with DMEM, 25 mM glucose, and 10% FBS until used in the experiments 10–14 days after initiation of the differentiation protocol, when between 80 and 90% of the cells exhibited the adipocyte phenotype. In differentiated adipocytes, various experiments involved stimulation with insulin (10μg/ml) for 30 min at 37°C.

### Glucose transport activity assays

For measurement of glucose transport activity, adipocyte cells or conditioned medium cultured cells (two days prior the assays) were washed three times with transport buffer (pH7.4), consisting of 20 mM HEPES, 120 mM NaCl, 1.2 mM MgSO_4_, 2 mM CaCl_2_, 2.5 mM KCl, 1 mM NaH_2_PO_4_, and 1 mM sodium pyruvate, and incubated in this buffer for an additional 30 min in the absence (basal) and presence of a insulin concentration (100 nM) at 37°C. Glucose transport was assayed in monolayers as initial rates of 2-deoxy glucose uptake as we have previously described [11]. In these experiments, the distribution space of radiolabeled L-glucose was used to correct for nonspecific carryover of radioactivity with the cells and uptake of hexose by simple diffusion.

### Western blot analysis

Adipocytes were harvested from the culture plates with cell lysis buffer (1 × PBS, 1% Nonidet P-40, 0.5% sodium deoxycholate, 0.1% SDS) containing freshly added protease inhibitor cocktail (Sigma). Twenty-five μg of protein per lane and known molecular weight markers from Bio-Rad (Hercules, CA) were separated by SDS-polyacrylamide gel electrophoresis. Proteins were electrophoretically transferred onto nitrocellulose membranes and incubated overnight at 4°C with blocking solution (5% nonfat milk in TBS). The blocked membranes were separately incubated with the anti-resistin, -TNFα, -IL-6, -IL-10 or -MCP-1 antibodies or the horseradish peroxidase (HRP)-conjugated V5 epitope antibody (1:1000 dilution with 1% nonfat milk in TBS) for 1 hour at room temperature, and washed three times with TBS buffer containing 0.1% Tween 20 for 15 minutes at room temperature with shaking. The second antibody conjugated with horseradish peroxidase (HRP) (Santa Cruz Biotechnology) against to the premier antibody was added, incubated, and washed as described above for the first antibody. Immunodetection analyses were accomplished using the Enhance Chemiluminescence Kit (New England Nuclear Life Science Products, Boston, MA).

ELISA assays of resistin in 3T3-L1 cell culture medium samples were analyzed using a resistin ELISA kit from LINCO Research (St. Charles, MO). The assays were performed according to the manufacture's protocol.

### Real time QPCR analysis

About 5 μg of the total RNA was converted to first strand cDNAs in 20 μl reactions using random primers (Gibco BRL). For each quantitative PCR experimental set, the target gene primers (see bellow) were utilized to amplify a 10-fold serial diluted gene products (10 to 10^12^) to make a linear standard curve for each individual target gene. For an endogenous standard, 18S rRNA primers (5'-AAT TTG ACT CAA CAC GGG AAA CCT CAC-3' and 5'-CAG ACA AAT CGC TCC ACC AAC TAA GAA C-3') were simultaneously used for amplification from 1 μl first strand cDNAs in each 20 μl reaction tube; this allowed for normalization of cDNA loading and relative mRNA units in each of reactions.

QPCRs were performed using a Mx3000P™ Real-Time PCR System and a Brilliant SYBR Green QPCR Master Mix buffer (Stratagene) containing 100 mM KCl, 40 mM Tris-HCl, 0.4 mM of each dNTP, 2.5 mM MgCl_2_, SYBR Green I, SureStart Taq DNA polymerase (50 units/ml) with hot start capability, and 20 nM of fluorescein for experimental plate well factor collection on the Mx3000P™ Real-Time PCR System. A passive diluted reference dye (ROX) was also added to each tube (30 nM) to compensate for non-PCR related variations in fluorescence. The concentrations of the gene primers for ALBP/aP2 (5'-TAC TGG GCC AGG AAT TTG AC-3' and 5'-GTG GAA GTG ACG CCT TTC AT-3'); PPARγ (5'-TTT TCA AGG GTG CCA GTT TC-3' and 5'-AAT CCT TGG CCC TCT GAG AT-3'); C/EBPα (5'-GCT GGA GTT GAC CAG TGA CA-3' and 5'-AAA CCA TCC TCT GGG TCT CC-3'); GLUT 4 (5'-GAT TCT GCT GCC CTT CTG TC-3' and 5'-ATT GGA CGC TCT CTC TCC AA-3') were 500 nM for each primer and 1 μl first strand cDNAs for each real-time QPCR reaction performed in a volume of 20 μl. The thermal cycling program was 3 min at 95°C for enzyme activation (allowing an automated hot start PCR), 45 cycles of denaturation for 30s at 95°C, 30s annealing at 60°C, and 30s extension at 72°C.

Melting curve analysis was performed to confirm the real-time QPCR products. The amplified products were denaturated and reannealed at different temperature points to detect their specific melting temperature.

### Statistics

Experimental results were shown as the mean ± se. Statistical analyses were performed by unpaired Students' T-test assuming unequal variance unless otherwise indicated. Significance was defined as the P < 0.05.

## Results

### Generation of recombinant resistin lentiviruses and lentiviral transduced stable cell lines

**Figure 1:**
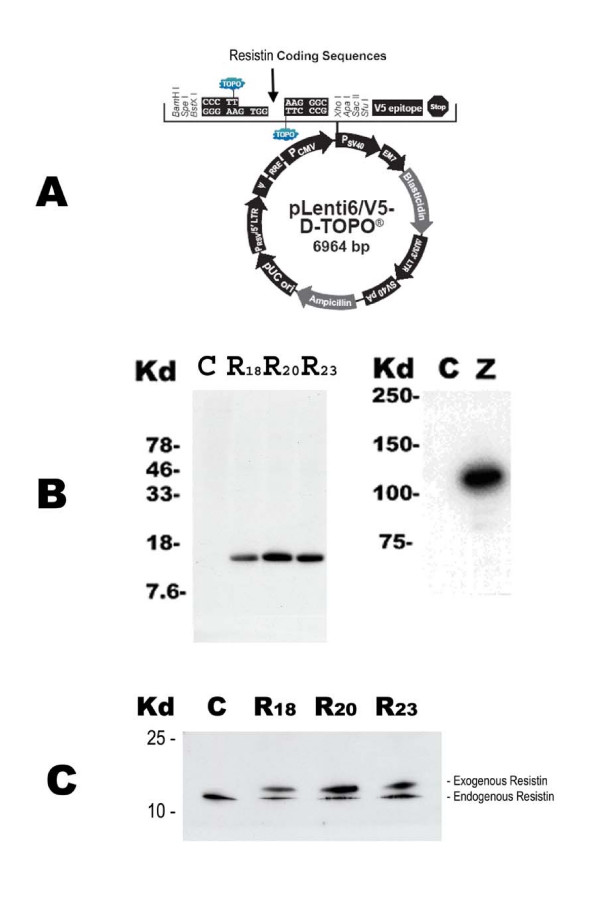
**Generation of recombinant resistin lentivirus and detection of recombinant gene expression. (A) **A full length of mouse resistin coding sequence (0.34 kb) was cloned into a ViraPower-CMV vector (Invitrogen). This recombinant resistin gene construct included a V5 epitope tag and a CMV promoter. **(B) **A western blot analysis was performed to confirm the expression of the recombinant resistin genes in three different stably transduced cell lines (R18, R20 and R23) using anti-V5 antibodies. The lanes marked by the letter C were loaded with proteins from non-transduced control 3T3-L1 cells. The lane marked by the letter Z was loaded with proteins from cells transduced lentivirus containing recombinant LacZ gene including the V-5 tag and LacZ was detected using anti-V5 antibodies. **(C) **A western blot analysis was performed to examine the secretion of the recombinant exogenous resistin proteins (R18, R20, and R23) and the endogenous resistin proteins in the medium of 3T3-L1 cells. Exogenous and endogenous resistin proteins were detected using anti-resistin antibodies. The control lane marked by the letter C was loaded with proteins from non-transduced 3T3-L1 cells.

To investigate effects of resistin on adipocytes, we stably transduced 3T3-L1 fibroblasts using lentiviral expression vectors. Full lengths of fusion cDNAs, including mouse resistin coding sequences (0.34 kb) and a V5 epitope tag, were cloned into a ViraPower-CMV vector (Invitrogen) (Fig. 1A). The recombinant lentiviral plasmids and a control lentiviral LacZ gene construct were transfected into HEK293 cells to generate the recombinant lentiviruses. X-gal staining was performed to confirm that the HEK293 cell transfection was successful and that infectious virus particles were produced (data not shown).

To establish stable 3T3-L1 fibroblast cell lines which overexpress resistin or LacZ genes, recombinant resistin or LacZ lentiviral stocks purified from HEK293 cells were used to infect 3T3-L1 fibroblasts. Forty-eight hours post-transduction, these cells were placed under blasticidin selection (10 μg/ml) for 20 days. The tests for stable recombinant resistin or LacZ gene expression were performed using an antibody against the V5 epitope tags in these recombinant proteins after antibiotic selection by Western blot analyses (Fig. 1B). We also examined the secretion of recombinant resistin proteins in the cell culture media from the selected resistin transduced cell lines after adipocyte differentiation (day 12, see below). Both endogenous native resistin as well as exogenous lentivirus-transduced resistin containing the V5 tag were readily detected in the media of fully differentiated adipocytes as shown in Figure 1C. Thus, cells transduced with the resistin lentivirus both synthesized and secreted resistin as a consequence of transgene introduction. Endogenous resistin levels in the media from these resistin transduced cell lines were comparable to levels observed in media from control cells. The overall secretion of resistin (endogenous + exogenous) was approximately 2–4 fold greater (95 ng/ml to 195 ng/ml by ELISA, data not shown) in the stable resistin-overexpressing cell lines compared with LacZ controls.

### Resistin hyperexpression in 3T3-L1 adipocytes

**Figure 2:**
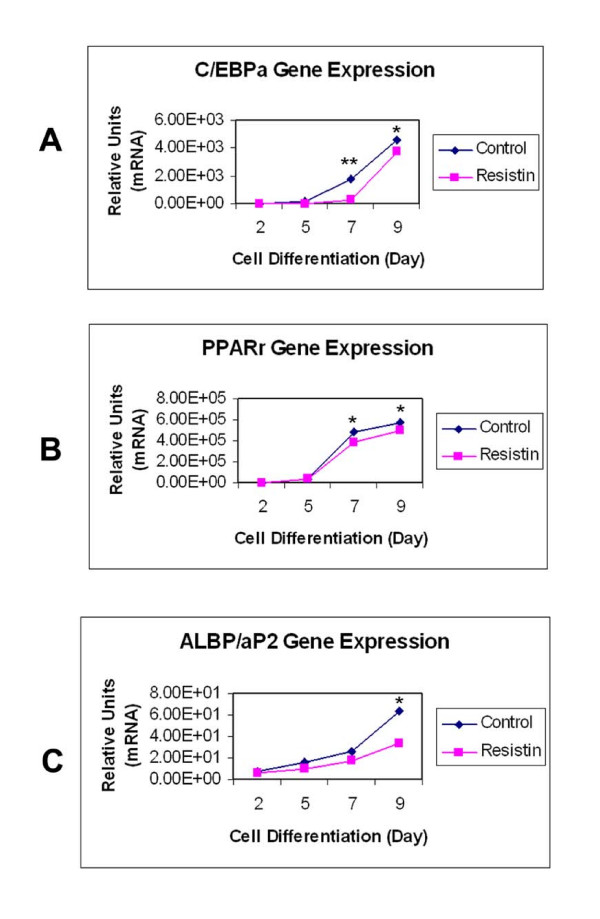
**Effects of resistin on gene expression patterns during adipogenesis**. Day 0 represents 3T3L1 fibroblasts reaching 100% confluence. Two days after full confluence (Day 2), cells were placed in DMEM containing 25 mM glucose, 0.5 mM isobutylmethylxanthine (IBMX), 1 μm dexamethasone (Dex), 10 μg/ml insulin, and 10% FBS for 3 days; then, on day 5 cells were placed in DMEM containing 25 mM glucose, 10 μg/ml insulin, and 10% FBS for 2 days. After day 7, cells were maintained in DMEM, 25 mM glucose, and 10% FBS. At the indicated time points, resistin and LacZ transduced preadipocytes and adipocytes were lysed and the mRNA levels encoding PPARγ, C/EBPα, and ALBP/aP2 were quantified by quantitative PCR. Results represent the mean ± SE from three separate experiments. Asterisk, P < 0.05; double asterisk, P < 0.01 for resistin versus control cells.

3T3-L1 fibroblasts, whether transduced with LacZ or resistin lentivirus, were differentiated into adipocytes under the standard induction protocol [12-14]. We studied adipogenesis in the LacZ control and resistin transduced adipocytes by examining the time-course of expression of two key transcriptional factors known to be involved in adipocyte differentiation [15], CCAAT/enhancer binding protein α (C/EBPα) and peroxisome proliferator-activated receptor gamma (PPARγ), as well as the adipocyte lipid binding protein (ALBP/aP2), which has been considered a hallmark of adipogenesis and is one of target genes for the PPARγ during adipocyte differentiation, [16,17]. To investigate whether overexpression of resistin would modify gene expression patterns, we measured mRNAs encoding these key regulators and marker during differentiation. As shown in Figure 2, expression of both C/EBPα and PPARγ was lower over days 5 to 9 during adipocyte differentiation in resistin overexpressing cells, than those in LacZ transduced controls. ALBP/aP2 expression was also reduced in the resistin overexpressing cells over this time period when compared with the controls. However, when we stained the adipocytes with Oil Red O to monitor the process of adipocyte differentiation, we did not detect significant differences in lipid content between the resistin transduced cells and the control cells (data not shown).

### Effects of resistin overexpression on insulin sensitivity and GLUT4 expression

**Figure 3:**
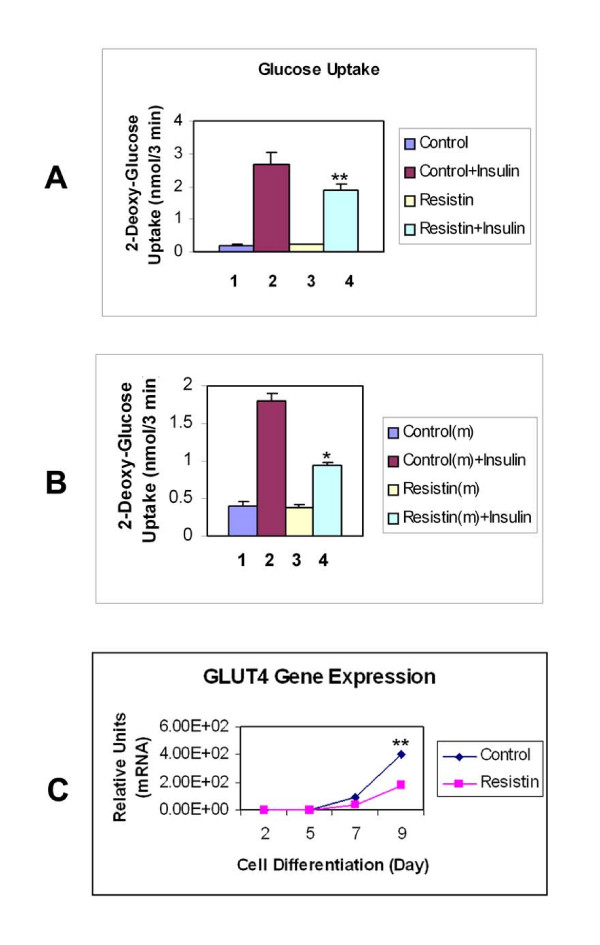
**Effects of resistin on GLUT4 activity and expression**. Adipocytes transduced with the LacZ gene (Control) or with the resistin gene were incubated for 12 days until fully-differentiated. **(A) Resistin overexpressing adipocytes show an inhibition of insulin-stimulated glucose transport activity**. Control cells (Bars 1 and 2) and cells over-expressing resistin (Bars 3 and 4), were incubated in the absence (Bars 1 and 3) or presence (Bars 2 and 4) of 100 nM insulin for 30 min at 37°C to induce maximal rates of glucose uptake. Double asterisk, P < 0.01 for (Resistin + Insulin) versus (Control + Insulin). **(B) Conditioned medium from resistin transduced adipocytes inhibits the activity of insulin-stimulated glucose transport**. 3T3-L1 adipocytes treated with conditioned control medium from LacZ transduced cells (control) (Bars 1 and 2) and conditioned medium from resistin transduced cells (Bars 3 and 4), were incubated in the absence (Bars 1 and 3) or presence (Bars 2 and 4) of 100 nM insulin for 30 min at 37°C to induce maximal rates of glucose uptake. Asterisk, P < 0.05 for (Resistin medium + Insulin) versus (Control medium + Insulin). **(C) Resistin decreases the gene expression of GLUT4**. Expression of glucose transporter GLUT4 gene was examined using a quantitative PCR assay in resistin transduced adipocytes and LacZ transduced adipocytes during their differentiation process as described in the Fig. 2. Results represent the mean ± SE from three separate experiments. Double asterisk, P < 0.01 for resistin versus control at Day 9.

Since resistin administration acutely impairs glucose tolerance and insulin action in skeletal muscle in animal models [8,18], next we examined whether resistin overexpression would affect the activity of insulin-stimulated glucose transport system in adipocytes. As shown by the data in Figure 3A, there was a 30% decrease (p < 0.01) in the maximal insulin-stimulated glucose transport rate in fully differentiated cells (day 12) over-expressing resistin (Bar 4, Resistin + Insulin) compared with LacZ-expressing control adipocytes (Bar 2, Control + Insulin). Similar effects of resistin overexpression were observed in three separate stably-transduced cell lines compared with LacZ expressing controls.

This effect on the insulin-stimulated glucose uptake was also observed when we used conditioned media from resistin-overexpressing cells versus media from LacZ-expressing cells to treat non-transduced 3T3-L1 adipocytes (Figure 3B). The adipocytes were cultured in conditioned media for two days prior the glucose transport assays. The maximal insulin-stimulated glucose transport rate was significantly decreased 50% (p < 0.05) in the cells cultured with the medium from resistin-transduced adipocytes (Bar 4, Resistin(m) + Insulin), compared to that from LacZ-transduced adipocytes (Bar 3, Control(m) + Insulin). Similar results were observed when using non-conditioned medium as a control; insulin-stimulated glucose transport was comparable in cells exposed to non-conditioned media and conditioned media from LacZ expressing cells, while the insulin action was reduced by 42% to 49% when cells were treated with conditioned media from resistin-overexpressing cells or purified resistin protein (data not shown).

To identify whether diminished activity of the insulin-responsive glucose transport system was associated with a decrease in GLUT4 gene expression, we measured GLUT 4 mRNA in both resistin overexpressing and control LacZ adipocytes. Figure 3C shows that GLUT4 mRNA levels were reduced by 51% in cells overexpression resistin compared with LacZ-transduced cells, and that the decline in GLUT4 mRNA levels was observed over days 5 to 9 during differentiation.

### Effects of resistin overexpression on cytokine production

**Figure 4:**
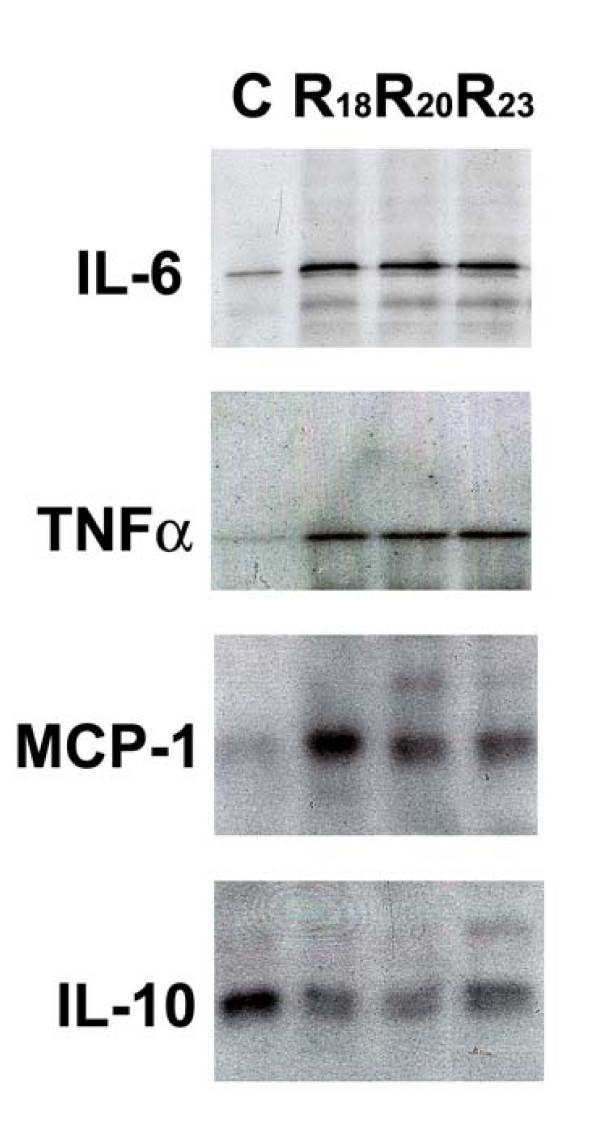
**Effects of resistin overexpression in adipocytes on proinflammatory and anti-inflammatory cytokines**. Western blot analyses were performed to examine the levels of three proinflammatory cytokines, IL-6, TNFα, and MCP-1, and one anti-inflammatory cytokine IL-10 in resistin transduced 3T3-L1 cell lines (R18, R20 and R23) and in the LacZ transduced control cells (C) which had been fully differentiated into adipocytes (Day 12).

Since the proinflammatory cytokines are known to exert effects on insulin action and substrate metabolism in adipocytes [19,20], we examined the impact of resistin overexpression on TNFα, IL-6, MCP-1 and IL-10 production. Clearly, there could exist interactions among these factors in the context of autocrine/paracrine regulation of adipocyte biology. Interestingly, transduction with the recombinant resistin lentivirus led to increased production of TNFα, IL-6 and MCP-1 proteins and conversely decreased production of IL-10 protein in fully differentiated adipocytes (Figure 4). Levels of TNFα, IL-6, and MCP-1 were increased by 10-, 3- and 21-fold (p < 0.05), in contrast, IL-10 was decreased by 2.5-fold in resistin overexpressing cells, respectively, compared with LacZ controls.

## Discussion

Resistin is one of multiple adipocytokines secreted by adipose tissue, and has been shown to modulate both glucose and lipid metabolism in vivo and in vitro. In animal models, it has been recently reported that mice with chronic hyperresistinemia have elevated glucose production due to the increased expression of hepatic phosphoenolpyruvate carboxykinase (PEPCK) and hepatic insulin resistance [9]. In L6 rat skeletal muscle cells, it has been shown that resistin does not alter insulin receptor signaling but does affect insulin-stimulated glucose uptake, presumably by decreasing the intrinsic activity of cell surface glucose transporters [21]. However, the mechanisms by which resistin could modulate insulin sensitivity remain obscure, and data addressing whether resistin can affect insulin action in adipocytes are lacking. The current studies demonstrate that overexpression of resistin in 3T3-L1 adipocytes, in conjunction with increased secretion of resistin into the surrounding media, are able to: (i) affect the expression pattern of key transcription factors during adipogenesis and, in fully differentiated cells, (ii) impair insulin's ability to stimulate glucose transport concomitant with a reduction in GLUT4 gene expression and (iii) augment production of inflammatory cytokines, TNFα and IL-6. These data indicate that resistin could function as a powerful autocrine/paracrine factor in adipose tissue regulating insulin action and adipocytokine production.

Adipogenesis from fibroblast precursors follows a highly ordered and well characterized temporal sequence. The first step is growth arrest of proliferating preadipocytes, followed by the coordinated sequential expression of key transcription factors that direct the adipogenic program. It has been shown that PPARγ and C/EBPα are key transcriptional factors for initiating adipocyte differentiation [22,23], and positively regulate each other's expression. However, PPARγ is felt to play a directorial role in the adipogenic hierarchy of transcription factors, while C/EBPα promotes specific aspects of the adipocyte phenotype including insulin sensitivity and lipid accumulation [15]. In the current study, resistin overexpression reduced mRNA levels for PPARγ and C/EBPα during adipogenesis, although no dramatic morphologic changes were observed in these cells during adipogenesis and lipid accumulation was not affected. Nevertheless, we did observe important functional defects in resistin overexpressing cells. PPARγ and C/EBPα direct the expression of a multiplicity of genes responsible for the fully-differentiated adipocyte phenotype, and two key functional proteins include ALBP/aP2 and GLUT4 [24]. With the expression of these proteins, cells acquire a capacity for insulin-stimulated glucose transport, and lipid droplets begin to appear in the cytoplasm and over time coalesce into fewer major droplets in the cells. The expression of both ALBP/aP2 mRNA and GLUT4 mRNA were suppressed in the resistin lentivirus-transduced cells compared with LacZ transduced controls. While the suppression of aP2 mRNA did not lead to measurable differences in cellular lipid content, the reduction in GLUT4 mRNA was accompanied by a marked attenuation in insulin stimulated glucose transport activity. Thus, resistin overexpression and hypersecretion induced insulin resistance in adipocytes. This effect of resistin overexpression was observed in three separate clones of stably-transduced cells and, therefore, appeared to be independent of the site of genomic integration.

Three proinflammatory cytokines produced by adipose tissue are TNFα, IL-6 and MCP-1. While these cytokines have been shown to modulate glucose homeostasis and lipid metabolism and to induce insulin resistance [19,20,25], the factors that regulate synthesis and secretion in adipose tissue are unknown. Our data demonstrated that resistin overexpression in adipocytes led to increased production of these proinflammatory cytokines. Interestingly, overexpression of resistin led to an inhibition of the production of IL-10, which limits inflammatory responses and promotes insulin sensitivity and is also down-regulated in metabolic syndrome [26]. These data are consistent with the idea that resistin secreted from adipocytes acted in an autocrine/paracrine manner to augment cytokine production. Regarding effects of resistin overexpression on insulin-stimulated glucose transport, we are unable to say whether this is due to a direct effect of resistin, or was secondarily caused by resistin-mediated hypersecretion of TNFα, IL-6 and MCP-1 or hyposecretion of IL-10.

Regardless of whether insulin resistance was induced directly by resistin or via increased secretion of cytokines, the data indicate that resistin, TNFα, IL-6 and MCP-1 are capable of functioning in an autocrine/paracrine manner to regulate adipocyte cell biology and proinflammatory cytokine secretion. Conditioned media from the resistin overexpressing cells was able to induce insulin resistance in LacZ transduced or non-transduced adipocytes, and contained only 2–4 times more resistin (from endogenous and transgene origin) than LacZ transduced control cells (endogenous origin only), which is within the physiological range of resistin production. Thus, the observed effects in cultured adipocytes could feasibly be relevant to events occurring in intact tissue. Our data, for the first time, demonstrate resistin's role in adipose tissue as a potential autocrine mediator of insulin resistance and inflammation that accompanies enlarging fat cell size and progressive obesity.

Adiponectin is another important adipocytokine that, in contradistinction to resistin, is secreted in lesser amounts with increments in fat cell size and acts to increase insulin sensitivity. For example, we have previously shown that adiponectin promotes adipogenesis, lipid accumulation, and insulin sensitivity when secreted into the media of cultured 3T3-L1 adipocytes [27]. Given the inverse relationship of adiponectin and resistin with respect to the influence of fat cell size on secretion and effects on insulin sensitivity, we examined whether overexpression of resistin or adiponectin would regulate each other in the transduced adipocytes. We observed that overexpression of either resistin or adiponectin did not regulate secretion of the other adipocytokine (data not shown). Therefore, while there could exist coordinate regulation of resistin and adiponectin, this will be mediated by a third factor that is independent of these adipocytokines and their action pathways.

## Competing interests

The author(s) declare that they have no competing interests.

## Authors' contributions

YF, LL and NL performed the experiments including data collection, statistical analysis, figure preparations. Both YF and WTG were responsible for review conception and design, interpretation, and critical revision of the manuscript.
